# iTRAQ-based quantitative proteomics analysis of cantaloupe (*Cucumis melo* var. saccharinus) after cold storage

**DOI:** 10.1186/s12864-020-06797-3

**Published:** 2020-06-03

**Authors:** Wen Song, Fengxian Tang, Wenchao Cai, Qin Zhang, Fake Zhou, Ming Ning, Huan Tian, Chunhui Shan

**Affiliations:** College of Food, Shihezi University, Xinjiang, 832000 China

**Keywords:** Cantaloupe, Cold storage, Proteomics, iTRAQ

## Abstract

**Background:**

Cantaloupe is susceptible to cold stress when it is stored at low temperatures, resulting in the loss of edible and commercial quality. To ascertain the molecular mechanisms of low temperatures resistance in cantaloupe, a cold-sensitive cultivar, Golden Empress-308 (GE) and a cold-tolerant cultivar, Jia Shi-310 (JS), were selected in parallel for iTRAQ quantitative proteomic analysis.

**Results:**

The two kinds of commercial cultivars were exposed to a temperature of 0.5 °C for 0, 12 and 24 days. We found that the cold-sensitive cultivar (GE) suffered more severe damage as the length of the cold treatment increased. Proteomic analysis of both cultivars indicated that the number of differentially expressed proteins (DEPs) changed remarkably during the chilly treatment. JS expressed cold-responsive proteins more rapidly and mobilized more groups of proteins than GE. Furthermore, metabolic analysis revealed that more amino acids were up-regulated in JS during the early phases of low temperatures stress. The DEPs we found were mainly related to carbohydrate and energy metabolism, structural proteins, reactive oxygen species scavenging, amino acids metabolism and signal transduction. The consequences of phenotype assays, metabolic analysis and q-PCR validation confirm the findings of the iTRAQ analysis.

**Conclusion:**

We found that the prompt response and mobilization of proteins in JS allowed it to maintain a higher level of cold tolerance than GE, and that the slower cold responses in GE may be a vital reason for the severe chilling injury commonly found in this cultivar. The candidate proteins we identified will form the basis of future studies and may improve our understanding of the mechanisms of cold tolerance in cantaloupe.

## Background

The cantaloupe (*Cucumis melo* var. saccharinus) is rich in various nutrients and is one of the main economic crops of Xinjiang, China, where it plays an important role in promoting local economic development [1]. Cantaloupe has a high sugar content and is susceptible to infection by pathogenic microorganisms [2], therefore, refrigerated storage is considered to be the most effective method for preserving the good quality of cantaloupe during long-distance transport [3]. However, cantaloupe is susceptible to cold damage during refrigeration, resulting in peel pitting and softening [4]. Use of cold-sensitive cultivars, longer storage times and lower temperatures are the major factors that contribute to the chilly injury.

As a major environmental stress, chilly stress affects plant growth and triggers a series of changes in many physiological and molecular processes [5]. It results in electrolyte leakage, accumulation of reactive oxygen species (ROS) including hydrogen peroxide (H_2_O_2_) and malondialdehyde (MDA), as well as changes in the levels of endogenous abscisic acid [6, 7], ethylene [8, 9] and soluble sugars [10] in plants. The accumulation of ROS may result in oxidative stress, which damages the plant plasma membrane, decreases enzyme activity, and inhibits the rates of photosynthesis and protein processing [11, 12]. Plant has evolved complex regulatory mechanisms to cope with cold stress. Stress-responsive signaling pathways regulate the expression levels of several downstream stress-related genes in response to low temperature stress [13]. Alongside this, non-enzymatic and enzymatic antioxidant systems participate in ROS scavenging to protect plant cells from oxidative damage [14].

To understand how plant copes with abiotic stresses, previous studies have employed physiological and transcriptomics approaches [4, 15]. Although transcriptome sequencing is a powerful method for identifying novel transcripts and analyzing gene expression at the transcriptional level, the changes in mRNA levels determined by transcriptomics do not always correlate with corresponding proteins changes [16]. In addition, the proteome of cantaloupe in response to cold remains largely unknown. In our study, we applied proteomics technology to directly visualize the proteins being expressed in cantaloupe.

To better investigate cold response mechanisms in cantaloupe over a period of time, we used iTRAQ coupled to liquid chromatography-tandem mass spectrometry (LC-MS/MS) to study two different cultivars (Golden Empress-308, GE; Jia Shi-310, JS) in parallel during cold treatments at 0.5 °C. We found that the cold damage began to appear at 12 days, and, by 24 days it was more obvious and severe. The findings of this study will lay a new foundation for the further investigation of cold tolerance mechanisms in cantaloupe, which may inform breeding programs and improve the commercial storage of this fruit.

## Results

### Phenotypic changes in GE and JS under cold treatment

**Fig. 1:**
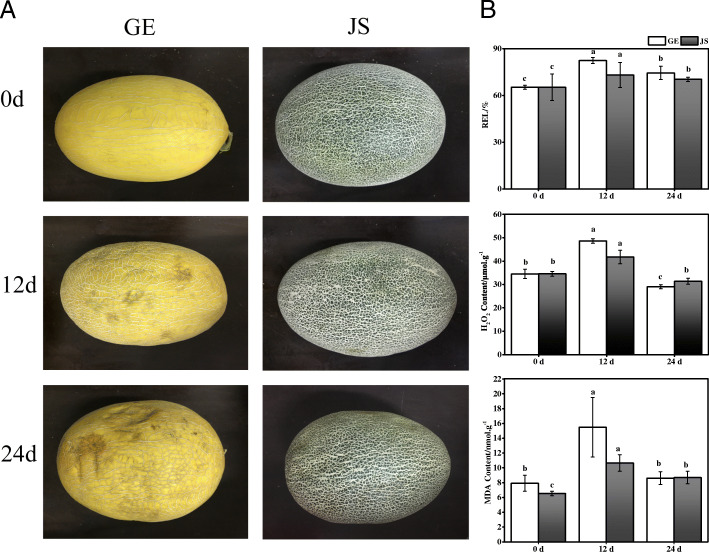
The phenotypic changes of GE and JS under cold treatment at different time points. **a**, Representative pictures of untreated GE and JS fruits, and fruits exposed to 12 or 24 d of cold treatment; **b**, Comparison of REL, MDA content and H_2_O_2_ content in GE and JS fruits after cold treatment with the control. The different letters represent that they are significantly different based on ANOVA (*P* < 0.05)

After 12 days of cold storage, we observed several phenotypic changes in GE, but not in JS. After 24 days, GE fruits were suffering more severe chilling injuries including rotting and bacterial infections, while JS fruits retained a higher quality with fewer injuries (Fig. 1a). Cold stress resulted in increases in relative electrolyte leakage (REL) in both cultivars during 12 days of storage, which was higher in GE than in JS, but the RELs of both cultivars decreased after 24 days of storage (Fig. 1b). Similar tendencies were also identified among the changes in malondialdeobhyde (MDA) and H_2_O_2_ levels (Fig. 1b). Overall, our analysis confirmed that cold-sensitive GE suffered a more severe cold damage, with higher levels of electrolyte leakage, lipid peroxidation, and H_2_O_2_ than JS during the early phase of cold storage.

### Identification and quantitation of DEPs by iTRAQ

We compared protein levels in JS and GE before and after the cold treatment to identify differentially expressed proteins (DEPs). Using iTRAQ labeling LC-MS/MS analysis, 5450 proteins were specifically identified from 107,101 LC-MS/MS spectra and 30,927 peptides in GE, and 5291 proteins were identified from 107,048 LC-MS/MS spectra and 29,829 peptides in JS (Additional file 1: Table S1).

**Fig. 2:**
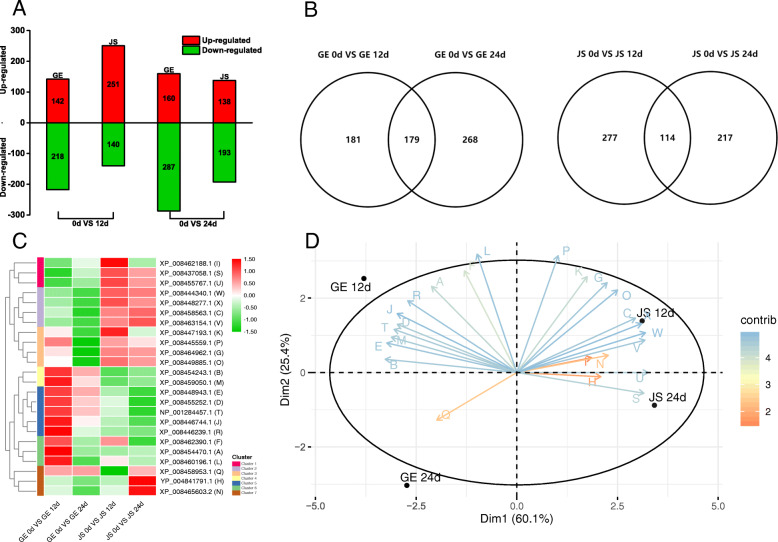
Differentially expressed proteins (DEPs) in GE and JS after 0, 12 and 24 days of cold treatment. **a**, The number of DEPs in GE and JS at different time points; **b**, Venn diagram of common and specific identified DEPs for GE and JS under cold treatment at different time points; **c**, Hierarchical clustering analysis of common expressed DEPs from GE and JS; **d**, Principal component analysis of common expressed DEPs from GE and JS

We used ratio fold changes of > 1.200 or < 0.833 in expression (*P* < 0.05) as the cut-off points for upregulated and downregulated proteins, respectively, and found a total of 807 DEPs (12 days: 360; 24 days: 447) in GE, and 722 DEPs (12 days: 391; 24 days: 331) in JS, after cold treatment (Additional file 2: Table S2). After 12 days of cold treatment, we identified 360 DEPs in GE, 142 of which were up-regulated and 218 of which were down-regulated (Fig. 2a). In JS, there were 391 DEPs, of which 251 were up-regulated and 140 were down-regulated (Fig. 2a). After 24 days, there were 447 DEPs in GE, 160 of which were up-regulated and 287 of which were down-regulated (Fig. 2a). Similarly, we found 331 DEPs in JS after 24 days, of which 138 were up-regulated and 193 were down-regulated (Fig. 2a). A higher number of DEPs were identified in JS than in GE at the early phase of cold treatment, but during the later phase of treatment, the number of DEPs in GE increased (Fig. 2a).

According to these observations, we assumed that the cold-tolerant cultivar JS responded faster than GE in terms of expressing cold-responsive proteins. Rapid up-regulation of proteins that regulate the response to the chilling stress and protect the plant cells from damage induced by ROS is important for cold tolerance. Therefore, the delayed cold response in GE may be a critical reason for the severe chilling injury [17]. Meanwhile, among the 807 DEPs in GE, 181 (22.42%) and 268 (33.20%) were specifically identified at the 12 and 24 day time points, respectively. A further 179 (22.18%) DEPs were shared by both time points (Fig. 2b). However, in JS, only 114 out of 722 DEPs (15.79%) were common to both time points, while 277 (38.37%) and 217 (30.06%) were specifically identified after 12 or 24 days, respectively (Fig. 2b). These findings demonstrate that much more different groups of proteins were mobilized in cold-tolerant JS under low temperature treatment [12].

In order to identify the proteins that are most likely to be related to the acquisition of cold tolerance in cantaloupe, a careful analysis of common expressed DEPs was carried out. The selected proteins with differential expression patterns were commonly expressed during the whole treatment period in both cultivars. A hierarchical cluster analysis (HCL) was performed to analyze the correlations of common expressed DEPs in GE and JS after cold treatment. Notably, the changes among 24 common DEPs were statistically significant and their abundance can be illustrated as seven clusters, revealing that two cultivars mobilized numerous proteins and differentially regulated their abundance to cope with cold stress (Fig. 2c; Additional file 2: Table S2). Furthermore, the principal component analysis (PCA) we performed on the expression data above indicated that, in all conditions, the two cultivars presented different protein expression patterns. The changes in protein expression between JS chilled at 12 and 24 days were smaller than those observed in GE, clustering close together with little separation in either axes (Fig. 2d; Additional file 2: Table S2). In contrast, the changes in protein expression in GE between 12 and 24 days clustered away from each other. Consequently, we speculated that, compared with JS, a longer duration of cold stress had a greater impact on the expression of proteins in GE, which may explain a more severe damage to GE during cold treatment [18].

### Primary functional classification of DEPs

**Fig. 3:**
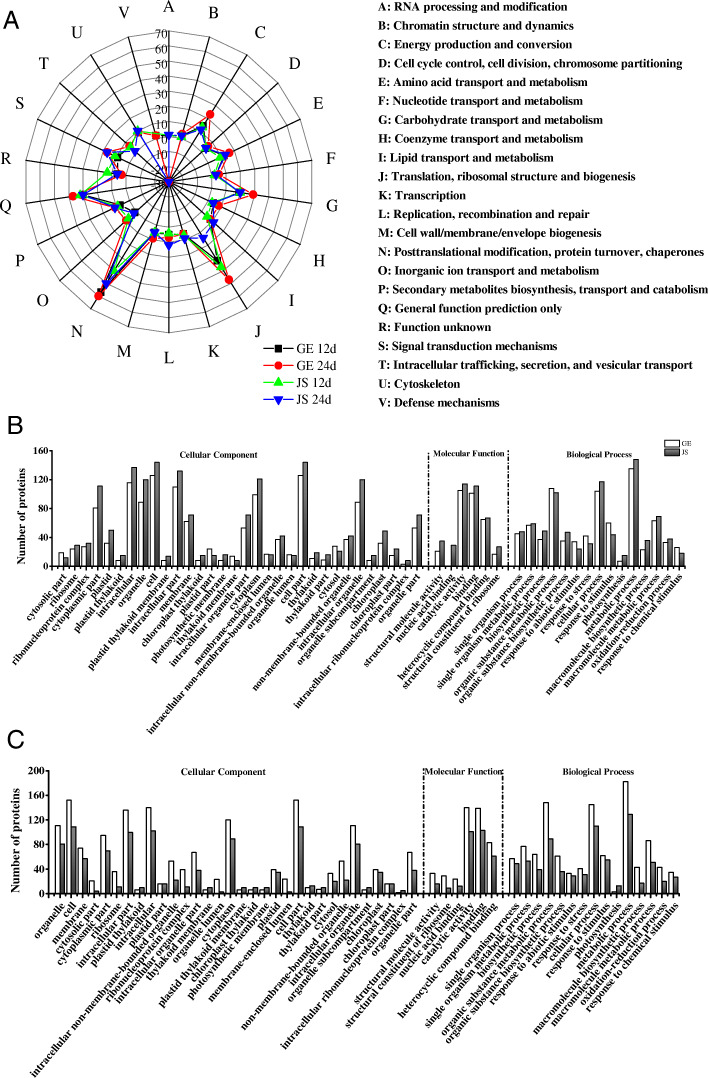
Function classification of the DEPs. **a**, COG function classification of DEPs in GE and JS; **b**, GO annotation of DEPs in GE and JS during the early phase of cold treatment; **c**, GO annotation of DEPs in GE and JS in the later phase of cold treatment

From the Clusters of Orthologous Groups (COG) database, we found that the largest group of DEPs are involved in ‘posttranslational modification, protein turnover, chaperones’ (119 DEPs), followed by ‘general function prediction only’ (94 DEPs) and ‘translation, ribosomal structure and biogenesis’ (83 DEPs; Fig. 3a; Additional files 3: Table S3). The further analysis will be discussed below.

### DEPs in response to the early phase of cold stress

Using gene ontology (GO) analysis, the DEPs were classified into three categories: cellular components (CC), molecular function (MF) and biological processes (BP) [19]. During the early phase of cold stress in both cultivars, the most highly represented categories were ‘cell’, ‘cell part’, ‘intracellular’, ‘intracellular part’ and ‘cytoplasm’ in CC (Fig. 3b); ‘catalytic activity’, ‘binding’ and ‘heterocyclic compound binding’ in MF (Fig. 3b); and ‘metabolic process’, ‘organic substance metabolic process’ and ‘cellular process’ in BP (Fig. 3b). The results indicated that the majority of DEPs were involved in metabolic processes, cellular processes, cell and catalytic activities, suggesting the cold treatment mainly affected physiological metabolism and cell differentiation in cantaloupe (Additional files 4: Table S4). More intriguingly, all three categories of proteins were expressed at higher levels in JS compared with GE, revealing that, the proteome of cold-tolerant JS responds more rapidly to cold stress than that of cold-sensitive GE.

**Fig. 4:**
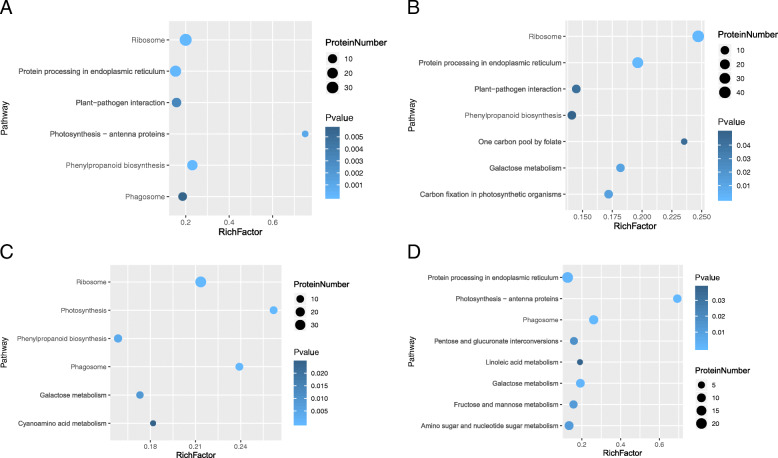
KEGG pathway enrichment. **a**, KEGG pathway enrichment in GE after 12 days of cold treatment; **b**, KEGG pathway enrichment in GE after 24 days; **c**, KEGG pathway enrichment in JS after 12 days; **d**, KEGG pathway enrichment in JS after 24 days

**Fig. 5:**
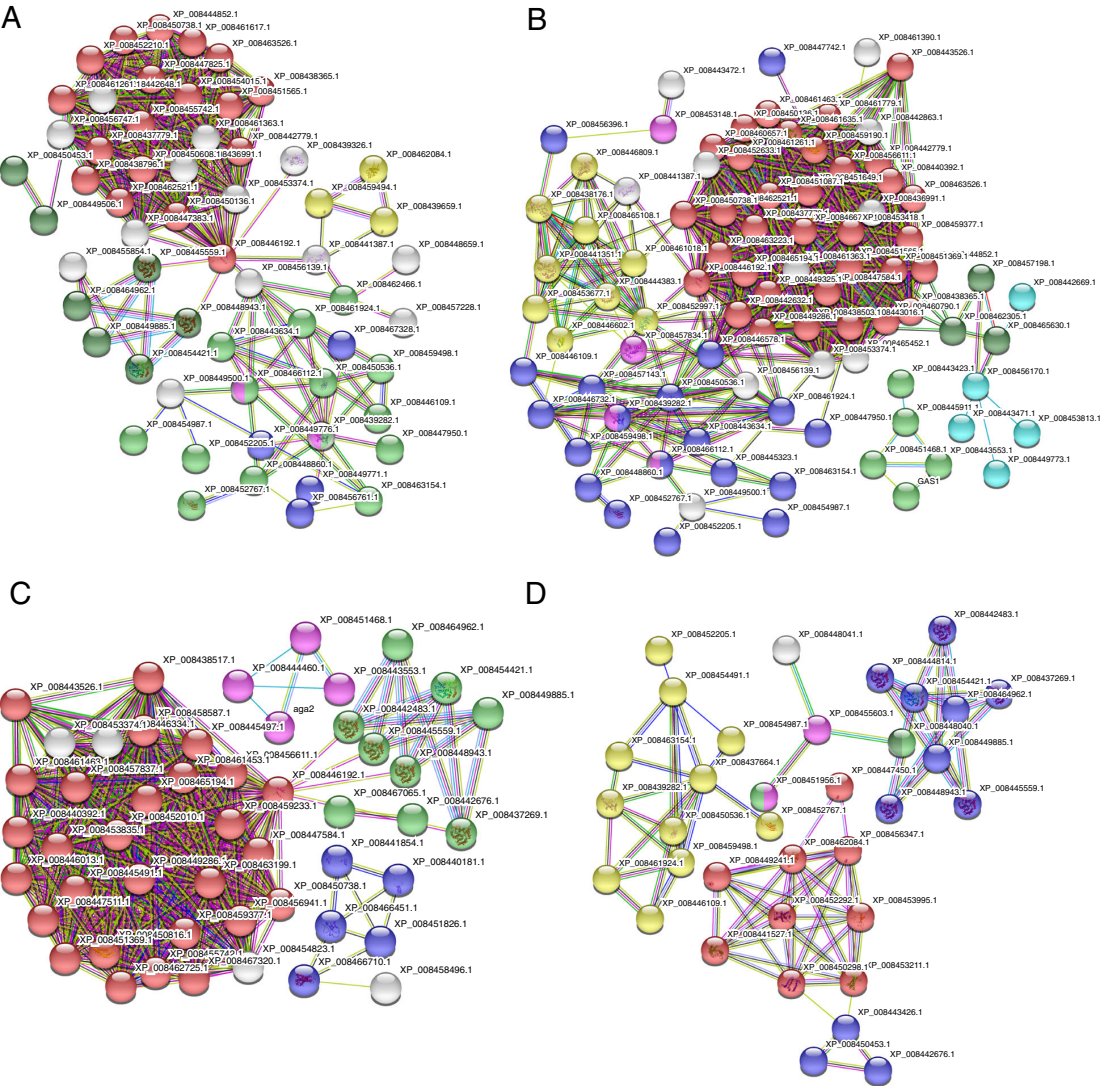
PPI analysis of DEPs based on KEGG pathway enrichment. **a**, PPI in GE after 12 days (Different node colors show the types of enrichment according to *P*-value: ribosome in red, phenylpropanoid biosynthesis in blue, protein processing in endoplasmic reticulum in green, photosynthesis-antenna proteins in yellow, plant–pathogen interactions in pink, phagosome in dark green); **b**, PPI in GE at 24 days (Different node colors show the types of enrichment according to *P*-value: ribosome in red, protein processing in endoplasmic reticulum in blue, galactose metabolism in green, carbon fixation in photosynthetic organisms in yellow, plant–pathogen interactions in pink, one carbon pool by folate in dark green, phenylpropanoid biosynthesis in light blue); **c**, PPI in JS after 12 days (Different node colors show the types of enrichment according to *P*-value: ribosome in red, photosynthesis in blue, phagosome in green, phenylpropanoid biosynthesis in yellow, galactose metabolism in pink); **d**, PPI in JS after 24 days (Different node colors show the types of enrichment according to *P*-value: photosynthesis-antenna proteins in red, phagosome in blue, galactose metabolism in green, protein processing in endoplasmic reticulum in yellow, amino sugar and nucleotide sugar metabolism in pink, fructose and mannose metabolism in dark green, linoleic acid metabolism in light blue). STRING tool (http://string.embl.de/) was used to predict protein–protein interaction networks

To further identify the roles of the DEPs, we performed KEGG pathway analysis. Only significantly enriched categories with *P*-values < 0.05 were selected. We found that cold stress affected ribosome, phagosome and phenylpropanoid biosynthesis in both cultivars. Proteins involved with protein processing in the endoplasmic reticulum, plant-pathogen interactions and photosynthesis-antennae were highly enriched in GE compared with JS. On the other hand, proteins involved in photosynthesis, galactose metabolism and cyanoamino acid metabolism, were considerably enriched in JS (Table 1; Fig. 4a, c; Additional files 6: Table S5). More interestingly, there were conspicuous protein-protein interactions among ribosome and other functions (*P* < 0.05) (Fig. 5a, c). Thus we speculate that ribosome related DEPs may play a significant role in regulating the metabolic mechanisms in cantaloupe at the early phases of cold stress.

**Table 1:** KEGG pathway analysis of proteins in GE and JS during chilling stress at different time period

Pathway name	Pathway ID	Number of proteins			
		GE		JS	
		12 d	24 d	12 d	24 d
Ribosome	ko03010	34	42	38	–
Phenylpropanoid biosynthesis	ko00940	18	11	13	–
Protein processing in endoplasmic reticulum	ko04141	25	32	–	21
Photosynthesis - antenna proteins	ko00196	3	–	–	9
Plant-pathogen interaction	ko04626	12	11	–	–
Galactose metabolism	ko00052	–	10	9	10
Carbon fixation in photosynthetic organisms	ko00710	–	11	–	–
One carbon pool by folate	ko00670	–	4	–	–
Photosynthesis	ko00195	–	–	11	–
Phagosome	ko04145	8	–	11	12
Cyanoamino acid metabolism	ko00460	–	–	6	–
Amino sugar and nucleotide sugar metabolism	ko00520	–	–	–	11
Fructose and mannose metabolism	ko00051	–	–	–	8
Pentose and glucuronate interconversions	ko00040	–	–	–	7
Linoleic acid metabolism	ko00591	–	–	–	4

### DEPs in response to the later phase of cold stress

As above, during the later phase of the cold stress, proteins were characterized by ‘cell’, ‘cell part’, ‘intracellular’, ‘intracellular part’ and ‘cytoplasm’ in CC (Fig. 3c); ‘catalytic activity’, ‘binding’ and ‘heterocyclic compound binding’ in MF (Fig. 3c); and ‘metabolic process’, ‘cellular process’ and ‘organic substance metabolic process’ in BP (Fig. 3c). After 12 days of cold storage, all three categories were dramatically higher in GE compared with JS, indicating that GE experienced greater levels of cold stress as the length of storage increased (Additional files 4: Table S4). The tardiness of the cold response in GE may be a critical reason for its severe cold damage.

KEGG pathway analysis indicated that protein processing in the endoplasmic reticulum and galactose metabolism may be affected in both cultivars after cold stress. Proteins involved in ribosomes, carbon fixation in photosynthetic organisms, plant-pathogen interactions, one carbon pool by folate, and phenylpropanoid biosynthesis were highly enriched in GE while proteins involved in photosynthesis-antennae, phagosomes, amino sugar and nucleotide sugar metabolism, fructose and mannose metabolism, pentose and glucuronate interconversions, and linoleic acid metabolism were enriched in JS (Table 1; Fig. 4b, d; Additional files 5: Table S5). Like the hallmark of the protein-protein interaction at 12 days, there was still a significant interaction among ribosome and other functions in GE, while dramatic changes happened in JS (Fig. 5b, d). This further demonstrates that various proteins were mobilized in JS during the cold treatment and that there was a positive relationship between the diversity of proteins and cold tolerance.

### Functional distribution analysis of cold induced proteins in JS

Based on GO analysis, functional distribution analysis were performed and the DEPs identified in cold-tolerant JS after 12 days cold treatment were selected as the cold induced proteins [20] (Additional files 4: Table S4; Additional files 6: Fig. S1). In terms of cellular components, membrane, cell part, cell, and organelle proteins were significantly enriched in JS (*P* < 0.05). In the molecular function category, proteins with catalytic activity and binding were the most positively regulated (*P* < 0.05). In biological processes, proteins involved in cellular processes, metabolic processes and organic substance metabolic processes were the most highly enriched (*P* < 0.05).

### Analysis of metabolites in response to cold stress

A limit of a 1.000-fold change coupled with a Student’s *t*-test (*P* < 0.05) was used to identify the differentially expressed metabolites. Metabolic data indicated that amino acids, such as proline, 3-hydroxy-L-proline 3 and 3-cyanoalanine, accumulated to higher levels during the whole period of cold storage in JS compared with GE (Table 2; Additional files 7: Table S6).

**Table 2:** Amino acids metabolites with changes in abundance (relative content) during chilling stress in GE and JS at different periods

Compound	GE		JS	
	12 d	24 d	12 d	24 d
Proline	1.400016	1.899369	3.005204	2.454753
3-hydroxy-L-proline 3	0.87722	1.185156	1.272809	1.571988
3-cyanoalanine	0.682412	1.342197	1.569883	1.087918

### Validation of DEPs by analysis of gene expression

**Fig. 6:**
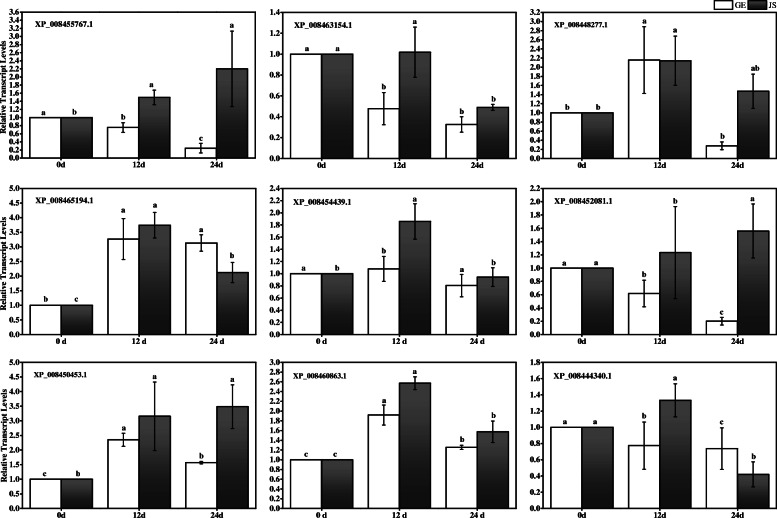
Relative quantification of the expression of eight genes in GE and JS after cold treatment. Statistical analyses were performed based on ANOVA, and different letters above the bars represent significance at *P* < 0.05

To validate the expression patterns of proteins obtained from the iTRAQ analysis, we randomly selected nine of the corresponding genes for q-PCR analysis using specific primers (Fig. 6; Additional files 8: Table S7). The results indicated that the expression patterns of five of the nine genes (55.56%) in GE and six of the nine genes (66.67%) in JS were consistent with the iTRAQ data, suggesting that these independent evaluations revealed a reliability of the iTRAQ data [21].

### Putative candidate proteins for cold tolerance in cantaloupe

In an attempt to identify proteins that may be involved in cold tolerance mechanisms in cantaloupe, we selected 258 and 247 proteins that showed differential expression among two cultivars. The selected candidate proteins were grouped according to the above phenotypic analysis and bioinformatics analysis as follows: carbohydrate and energy metabolism, stress responses, structural proteins, amino acid metabolism and signal transduction (Additional files 9: Table S8). Their expression patterns and possible roles will be discussed below.

## Discussion

### Proteins related to carbohydrate and energy metabolism

Metabolism is tightly associated with physiological adaptations in plants during stress responses [22]. Photosynthesis is a fundamental metabolic process for plant growth and development and is very sensitive to cold stress [23]. The chloroplast is an important organelle for photosynthesis in plant cells and recent studies showed that the chloroplast also participates in responses to environmental stresses [24, 25]. Chlorophyll a/b-binding proteins are affected by various environmental stresses. They are associated primarily with chlorophylls and xanthophylls, which serve as antenna complexes that absorb sunlight and transfer the excitation energy to the core complexes of photosystem II to drive photosynthetic electron transport [26, 27].

Our study indicated that the expression of chlorophyll a/b-binding proteins was inhibited by cold stress, however, many more of them were down-regulated in GE than in JS. After 12 days of cold treatment, three out of 13 chlorophyll a/b-binding proteins (XP_008462084.1, XP_008459494.1, XP_008439659.2) were down-regulated in GE, whereas only two were down-regulated in JS. After 24 days, two of the chlorophyll a/b-binding proteins were still down-regulated in GE, while nine were up-regulated in JS.

Xianfeng et al. [28] found that the expression of four chlorophyll a/b-binding proteins (Cla019105, Cla022963, Cla011748, and Cla013826) increased in cold-tolerant self-grafted (SG) watermelon seedlings, whereas no changes in expression were observed in cold-sensitive pumpkin rootstock-grafted (RG) watermelon seedlings. Our results confirmed that JS had a higher photosynthetic rate than GE at low temperatures. As a major contributor to cold acclimation, carbohydrate metabolites act as cryoprotectants and ROS scavengers along with their primary roles in photosynthesis [29]. Numerous studies have shown positive correlations between carbohydrate metabolism and the degree of cold tolerance in plants [30, 31]. Based on the COG analysis mentioned above, seven out of 37 carbohydrate-metabolism-related proteins were up-regulated, and 12 were down-regulated, in GE at the early phase of cold storage. In JS, however, 10 of 35 were up-regulated and 10 were down-regulated (Additional file 9: Table S8). Many more proteins were up-regulated in JS than in GE, suggesting carbohydrate-metabolism-related proteins may be essential for cold stress adaptation, and overexpression of carbohydrate metabolism in early cold treatment may provide sufficient energy production in JS to overcome cold stress.

The plasma membrane is the major ion pump of cells. It plays a central role in plant nutrition and growth. In all living organisms, lipid bilayer membranes constitute chemical barriers to the environment and, in eukaryotes, between organelles [32]. Under cold stress, the plasma membrane is the primary site for freezing injury in plants, which disturbs metal ions, metabolites, nutrient exchange and the regulation of cellular processes [33]. This in turn affects the normal growth and metabolism of plants.

We found 42 (21 up-regulated, 21 down-regulated) and 38 (10 up-regulated, 28 down-regulated) membrane-related DEPs in the early phase of cold storage in GE and JS, respectively (Additional files 9: Table S8). During the later phase, 52 (37 up-regulated, 15 down-regulated) and 31 (9 up-regulated, 22 down-regulated) DEPs were identified in GE and JS, respectively (Additional files 9: Table S8). The results revealed that the cold treatment affected the membranes of both cultivars. However, many more membrane proteins were affected by the cold treatment in GE than in JS, leading to more severe damage observed in GE. We also identified three DEPs (XP_008459785.1, XP_008451480.1, XP_016900197.1) that were specifically up-regulated in JS.

One of the membrane proteins we identified was an ATP synthase, which produces ATP from ADP in the presence of a proton gradient across the membrane and has a positive role in cold resistance [34, 35]. The V-type ATPase is one of the key proteins in maintaining the ion homeostasis inside plant cells [36]. The V-type proton ATPase subunit D (XP_008449506.1) and V-type proton ATPase subunit F (XP_008450453.1) remained unchanged in JS, but they were down-regulated in GE. Moreover, after 12 days, 2 ATPase-related proteins (XP_008442454.1, XP_008446865.1) were up-regulated in JS, but their expression remained unchanged in GE. Consequently, it has been speculated that JS has a greater ability to produce energy than GE, which may help plants to cope with low temperatures.

### Stress-related proteins

The oxidative burst, which is caused by the generation of large quantities of ROS, may cause cell death. Glutathione S-transferase (GST) plays important roles in oxidative stress tolerance, cellular detoxification and antioxidant defenses, and its stress-response mechanisms have been investigated in sweet potato [37]. In our study, we identified a total of 27 GST-related DEPs in GE and JS. The Tau and Phi classes of GSTs are the most common in cantaloupe and most were up-regulated in both GE and JS, but more members were up-regulated in JS than in GE. In particular, glutathione S-transferase zeta class-like (XP_008460863.1) and glutathione S-transferase-like (XP_008451606.1) were up-regulated in JS during the whole period of cold storage. Thus, we speculated that the continuous up-regulation of GSTs promotes homeostasis of ROS scavenging and increases the tolerance of cantaloupe to low temperatures.

Peroxiredoxin contributes to the stability of macromolecules under cold stress [38]. Our results showed that two thioredoxins (XP_008445916.1, XP_016902770.1) were specifically up-regulated in JS after 12 days of cold storage. We also identified three DEPs (XP_008448277.1, XP_008457297.1, XP_016901582.1) that were specifically up-regulated in JS in the early phase of treatment. The above results may partly explain why JS has greater cold-tolerance than GE, as confirmed by the data analysis of phenotypes shown in Fig. 1b.

### Structural proteins

The functional analysis of DEPs in cantaloupe revealed that proteins involved in ribosomes and protein processing in the endoplasmic reticulum were highly enriched in both cultivars under cold treatment. Numerous studies have indicated that ribosomal proteins [39], endoplasmic reticulum related proteins [40] and molecular chaperones involved in protein folding [41] are essential for protein synthesis, and they have been speculated to play central roles in regulating stress tolerance in plants. In this study, 38 ribosome-related proteins were found in GE after 12 days of cold treatment, of which 21 were down-regulated and three (XP_008447825.1, XP_008447383.1, XP_008453374.1) were up-regulated. In contrast, one out of 42 ribosome-related proteins were down-regulated, and 31 were up-regulated, in JS. After 24 days, 10 out of 38 ribosome-elated proteins remained unchanged, while 27 out of 38 proteins were down-regulated, in GE. However, only three out of 42 ribosome-related proteins were down-regulated, while two (XP_008445497.1, XP_008458587.1) were up-regulated, in JS. We hypothesized that the high expression of ribosome-related proteins at the early phase of cold storage may make JS synthesize and translate the relative proteins immediately to cope with chilling stress, which may contribute to the superior cold tolerance of JS compared with GE.

By regulating protein folding, stability, solubility, biogenesis and enzymatic activity, and preventing proteins from proteolytic degradation, the endoplasmic reticulum may mitigate the damage caused by the accumulation of misfolded proteins under low temperatures and other environmental stresses [42]. We found that 24 proteins involved with protein processing were down-regulated in the endoplasmic reticulum of GE in the early phase of the cold treatment. On the other hand, the expression of 21 of these proteins remained unchanged, and two (XP_008457228.1, XP_008463154.1) were up-regulated, in JS over the same period. Later on, 30 out of 32 DEPs were down-regulated, and two were up-regulated, in GE. In JS, however, only three were down-regulated, while 18 DEPs were up-regulated. This observation suggests that the up-regulation of endoplasmic-reticulum-related proteins enhanced the tolerance of JS as the cold storage continued.

DEPs related to protein folding act as molecular chaperones and are involved in various stress responses [43]. Widely known to be involved in plant responses to oxidative stress, heat shock proteins (HSPs) have also been shown to facilitate plant adaptation to environmental changes [44]. In our proteomic study, 15 out of 20 HSPs in GE were down-regulated after 12 days of cold treatment, whereas the expression of four (XP_008448853.1, XP_008448861.1, XP_008463154.1, XP_008463443.1) out of 25 HSPs in JS remained high. After 24 days, 19 HSPs were down-regulated in GE, while 19 were up-regulated in JS, which supports the idea that continuous high levels of HSPs may help plants adapt to stress stimuli [45]. DnaJ protein and Chaperonin have also been reported to protect plants against biotic and abiotic stress [46, 47]. Herein, chaperone protein Dnaj 10 isoform X2 (XP_008453046.1) was specifically up-regulated in JS after 12 days of cold treatment. Protein GrpE (XP_008455767.1) was also specifically up-regulated in JS. This result showed that the up-regulation of molecular chaperones may mobilize protein processing to enhance the cold tolerance of JS under cold stress.

### Amino acids metabolism related proteins

Metabolic adaptation is crucial for abiotic stress resistance in plants, and the accumulation of specific amino acids as well as secondary metabolites derived from amino acid metabolism has been implicated in increased tolerance to adverse environmental conditions [48, 49]. As a compatible osmolyte, proline has been shown to exert several adaptive functions under stress including stabilizing membranes and proteins, acting as a radical scavenger and providing carbon, nitrogen and energy for recovery after the stress [50]. Enhanced proline biosynthesis under stress has also been proposed to balance the redox status of the cell by maintaining a favorable NADP+/NADPH ratio [51]. In this work, one proline-related protein (XP_008454439.1) was up-regulated in JS, while its expression remained unchanged in GE. Our study also suggested that three cyanoamino acid relevant proteins (NP_001303611.1, XP_008452081.1, XP_016899729.1) were up-regulated in JS, which is in accordance with the findings of Yue et al. [52] (Table 2).

### Proteins related to signal transduction

The 14–3-3 proteins (14–3-3 s) could interact with other proteins and mediate diverse signaling pathways regulating many biological processes, such as metabolism, light and hormone signaling [53]. We found that, after 12 days of cold treatment, 14–3-3 protein 7-like (XP_008444837.1) was up-regulated in JS, but its expression remained low in GE. This suggests that the up-regulation of 14–3-3 s may play vital roles in the ability of cantaloupe to resist cold stress.

Ca^2+^-signaling-related proteins are important regulators of transcription, posttranscriptional processes and different metabolic functions in dicotyledonous species [54]. In our study, we found four Ca^2+^-signaling-related proteins (XP_008444340.1, XP_008448907.1, XP_008457834.1, XP_008453475.1) were specifically up-regulated in JS during the early phase of cold treatment. The results indicated that these Ca^2+^-signaling related proteins may promote cold tolerance in JS. Our study also identified two serine/threonine-protein kinases in GE that were down-regulated (XP_008458707.1, XP_008441406.1) during the early phase of cold treatment, while the expression of two serine/threonine-protein kinases (XP_008442585.1, XP_008451160.1) in JS remained high. We found uncharacterized protein C167.05 (XP_008442491.1), probable protein phosphatase 2C 39 (XP_008463673.1), protein BOLA2 (XP_008460912.1), uncharacterized protein LOC103500783 isoform X2 (XP_016902839.1) and universal stress protein A-like protein (XP_008463151.1) were specifically up-regulated in JS. The mechanisms by which these five proteins are regulated remain to be further studied in the future.

## Conclusions

We used iTRAQ-based proteomic analysis to compare the cold-tolerant cultivar JS with its cold-sensitive recurrent parent, GE, at two time points during cold storage. Physiological data indicated that both GE and JS began to accumulate ROS early in the chilling treatment. The expression levels of several significant categories of proteins, including carbohydrate and energy metabolism, stress-response-related proteins, structural proteins, amino acid metabolism, and signal transduction were found to change in both cultivars during the cold treatment. Metabolic analysis indicated that more factors were up-regulated in JS than in GE after 12 days. The prompt response and dramatic mobilization of proteins in JS allowed this cultivar to maintain a higher level of energy metabolism that enhanced the synthesis and degradation of proteins, and increased the ROS scavenging capacity, resulting in a higher level of cold tolerance when compared with GE. In addition, several candidate proteins like glutathione S-transferase, ribosomal proteins, heat shock proteins, proline iminopeptidase and calcyclin-binding protein may promote cold tolerance. These candidate proteins could be further studied in the future. Furthermore, studies that combine proteomics with metabolomics and physiological analysis may illuminate and deepen our understanding of the mechanisms that underlie chilling stress tolerance in cantaloupe.

## Methods

### Plant materials and treatment conditions

Two commercial varieties of cantaloupe (*Cucumis melo* var. saccharinus) Golden Empress-308 (GE) and Jia Shi-310 (JS) were collected from No. 121 Regiment farm in Shihezi, Xinjiang, China. They were identified by the Processing and Storage of Fruits & Vegetables Institute, Shihezi University, Xinjiang, China. No other permissions were necessary to select the samples. After harvest, the fruits of uniform size were stored in chambers at 0.5 °C (0 ± 0.5 °C) for 0 (control), 12 and 24 days, respectively. The fruits were divided into three replicates per cultivar, each consisting of six fruits of similar size [18]. After storage, electrolyte leakage, H_2_O_2_ content and lipid peroxidation were measured, and the exocarp tissue of each replicate was stored immediately at − 80 °C. Three biological replicates were performed at each time point.

### The determination of chilling stress induced physiological changes

MDA content, lipid peroxidation and H_2_O_2_ content were determined according to the methods described by Carvajal [18]. Relative electrolyte leakage (REL) was measured as described [55].

### Protein extraction, quantification and digestion

To extract proteins from the samples, 1 to 2 g of each sample was weighed and placed in a mortar with 10% PVPP, and then was ground into powder with liquid nitrogen. After grinding, the sample was transferred to a 50 ml round bottom centrifuge tube with 5-fold volume of Lysis Buffer 3 (100 mM TRIS, pH 7.5, 2 mM EDTA, 2 mM EGTA, 5 mM beta-glycerophosphate, 20 mM sodium pyrophosphate, 0.1% Triton X-100, 0.1% Tween 20 and 0.1% Hydorol M), 1 mM PMSF and 2 mM EDTA. After being well-mixed, the sample was placed on ice for 5 min, and then soaked for 5 min in an ice bath (2 s/3 s) with a final concentration of 10 mM DTT. After centrifugation, an appropriate amount of Lysis Buffer 3 was added to the supernatant and left for 5 min to soak (2 s/3 s). The sample was then centrifuged at 25,000 g at 4 °C and placed in an ice bath for 20 min [12]. The supernatant was used for further experiments.

The Bradford protein assay was used for the protein quantification. Standard proteins (0.2 μg/μl BSA) were sequentially added to 96-well microtiter plates (wells A1 to A10), followed by the addition of pure water, and then 180 μl of Coomassie Brilliant Blue G^− 250^ Quantitative Working Solution was added to each well. After calculating the protein concentration of each sample, SDS-PAGE was carried out according to a previous study [56]. After electrophoresis, the proteins were stained for 2 h with Coomassie brilliant blue, and then the appropriate volume of decoloring solution (40% ethanol 10% acetic acid) was added to shaker decolorization solution. After digestion, the enzymatic peptides were subjected to desalting using a Strata X column and vacuum-dried.

### Peptide labeling and separation

Each tube of IBT (2 mg) reagent was dissolved in 80 μl of isopropanol and shaken to fully dissolve the powder. The digested and desalted peptide was dissolved in 0.2 M tetraethylammonium bromide (TEAB) solution to a concentration of 4 μg/μl, and shaken for more than 1 min to fully dissolve the peptides. Next, 100 μg of well-mixed peptides and 80 μl of IBT reagent with a pH 7.0–8.0 were shaken and centrifuged. The peptides from samples GE 0 d, GE 12 d, GE 24 d, JS 0 d, JS 12 d, and JS 24 d were labeled with tags 113, 114, 115, 116, 117, and 118, respectively. All labeled samples were multiplexed and vacuum-dried.

The label was added and allowed to stand at room temperature for 2 h to ensure the proteins were fully labeled. The Shimadzu LC-20AB liquid phase system was used to separate the peptides, with 5 μm of each sample being added to a 4.6 × 250 mm Gemini C18 column [12]. The extracted peptides were reconstituted with 2 mL of mobile phase A (5% ACN pH 9.8) and then injected and eluted at a flow rate gradient of 1 mL/min: 5% mobile phase B (95% CAN, pH 9.8) for 10 min, 5 to 35% mobile phase B for 40 min, 35 to 95% mobile phase B for 1 min, mobile phase B for 3 min, and 5% mobile phase B for 10 min [57]. The elution peak was monitored at a wavelength of 214 nm and one component was collected per minute. The samples were combined with a chromatographic elution peak map to obtain 20 components, which were then freeze-dried [57].

### LC-MS/MS analysis and data analysis

The dried peptide samples were reconstituted with mobile phase A (2% ACN, 0.1% FA), centrifuged at 20,000 g for 10 min, and the supernatant was taken for injection. Separation was carried out by a Shimadzu LC-20 AD model nanoliter liquid chromatograph. The sample was first enriched and desalted in a trap column, and then connected in series with a self-packed C18 column (75 μm inner diameter, 3.6 μm column particle size, 15 cm column length) and separated at a flow rate of 300 nl/min through the following effective gradient: 0–8 min, 5% mobile phase B (98% ACN, 0.1% FA); 8–43 min, the mobile phase B linearly increased from 8 to 35%; 43–48 min, the mobile phase B increased from 35 to 60%; 48–50 min, mobile phase B increased from 60 to 80%; 50–55 min, 80% mobile phase B; 55–65 min, 5% mobile phase B [57].

The peptides separated by liquid phase were ionized by a nanoESI source and then passed to a Q-Exactive tandem mass spectrometer (Thermo Fisher Scientific, San Jose, CA) for DDA (data dependent acquisition) mode detection. The main parameter settings were: 1.6 kV for the source voltage; 350–1500 m/z for the scan range of the high-level mass spectrum; 100 for the initial m/z the high-level mass spectrum is fixed to; 30,000 for the resolution. Higher collision energy dissociation (HCD) was performed at a collision energy of 30, and fragmentation was detected in Orbitrap, an AGC target value of 3–6 and dynamic exclusion of 30 s.

### Protein identification and bioinformatics analysis

The raw LC-MS/MS data were processed for database searching using Proteome Discoverer (version 1.3, Thermo Electron, San Jose, CA) and then analyzed by Mascot (version 2.3.02 Matrix Science, London, UK), which was set up to search against the NCBI database (www.ncbi.nlm.nih.gov/genome/10697). The following parameters were set: Ms./Ms. Ion search as the type of search, Trypsin enzyme, fragment mass tolerance of 0.05 Da, peptide mass tolerance of 20 ppm, Monoisotopic as the Mass Values, Oxidation (M) and IBT 10plex (Y) as variable modifications, and Carbamidomethyl (C), IBT 10plex (N-term) and IBT 10plex (K) as fixed modifications. The picked protein strategy was used to determine the false discovery rate (FDR) for peptide and protein identification [58]. Peptide identifications were accepted if their FDR value was < 1.0%, while protein identifications contained at least one unique peptide. To identify statistically significant DEPs, the relative fold change (RFC) of the proteins was determined by the ratio in the treated samples and controls according to a previously described method [59]. The RFC of proteins in was calculated as the ratio of GE 12d_114/GE 0d_113 or JS 12d_117/JS 0d_116 in the early phase of cold stress, and as GE 24d_115/GE 0d_113 or JS 24d_118/JS 0d_116 in the later phase (Additional file 2: Table S2). Protein quantification required a *P* < 0.05, and only fold-change ratios > 1.200 or < 0.833 were considered statistically significant [16, 28].

The Clusters of Orthologous Groups (COG) (http://www.ncbi.nlm.nih.gov/COG/) database and GO (Gene Ontology) annotation were assigned according to those reported in the NCBI (http://www.ncbi.nlm.nih.gov/gene) and Swissprot databases to functionally classify the DEPs. Pathway analyses of identified proteins were performed using the Kyoto Encyclopedia of Genes and Genomes (KEGG) database (http://www.genome.jp/kegg/) [16].

### Metabolite measurements

Gas chromatography/mass spectrometry (GC/MS) was optimized for amino acids according to Kind T and Dunn WB [60, 61]. For each freeze-dried protein sample, 10 mg was transferred into a 2 mL tube, and 450 μL pre-cold extraction mixture (methanol/dH2O (v:v) = 3:1) containing 10 μL internal standard (adonitol, 0.5 mg/mL stock) were added [62]. Samples were vortexed for 30 s and homogenized with a ball mill for 4 min at 35 Hz, followed by ultrasonication for 5 min in ice water [62]. After centrifugation at 4 °C for 15 min at 10,000 rpm, 100 μL of supernatant was transferred to a fresh tube [62]. To prepare the QC (quality control) sample, 50 μL of each sample was taken out and combined together [62]. After evaporation in a vacuum concentrator, 100 μL of Methoxyamination hydrochloride (20 mg/mL in pyridine) was added and then incubated at 80 °C for 30 min, then derivatized by 120 μL of BSTFA regent (1% TMCS, v/v) at 70 °C for 1.5 h [62]. The samples were gradually cooled to room temperature and 5 μL of FAMEs (in chloroform) was added to all the QC samples [63]. All samples were then analyzed by gas chromatograph coupled with a time-of-flight mass spectrometer (GC-TOF-MS) [60, 61].

GC-TOF-MS analysis was performed using an Agilent 7890 gas chromatograph coupled with a time-of-flight mass spectrometer [64]. The system utilized a DB-5MS capillary column. A 1 μL aliquot of sample was injected in splitless mode. Helium was used as the carrier gas, the front inlet purge flow was 3 mL min^− 1^, and the gas flow rate through the column was 1 mL min^− 1^ [64]. The initial temperature was kept at 50 °C for 1 min, then raised to 310 °C at a rate of 10°Cmin^− 1^, then kept for 8 min at 310 °C [64]. The injection, transfer line, and ion source temperatures were 280, 280 and 250 °C, respectively [64]. The energy was − 70 eV in electron impact mode. The mass spectrometry data were acquired in full-scan mode with the m/z range of 50–500 at a rate of 12.5 spectra per second after a solvent delay of 6.25 min [64].

Raw data analysis were finished with Chroma TOF (V 4.3x, LECO) software and the LECO-Fiehn Rtx5 database was used for metabolite identification by matching the mass spectrum and retention index [62]. Finally, the peaks detected in less than half of the QC samples or RSD>30% in QC samples were removed [62]. Six biological replicates were collected for each cultivar at every time point and significant enrichment was detected at *P* < 0.05. Data analysis was carried out as previously described [49].

### Quantitative real time reverse transcription PCR analysis

Total RNA extraction, DNase treatment and cDNA synthesis were performed as previously described [4]. The relative expression of each target gene was calculated by using the 2^−ΔΔCt^ method and GAPDH gene was used as the internal reference gene for the normalization of all Ct values (Additional files 8: Table S7). Values were presented as the mean of three independent analyses.

## Supplementary information

**Additional file 1: Table S1.** Proteins in GE and JS identified by iTRAQ labeling.

**Additional file 2: Table S2.** Identified DEPs from GE and JS after cold treatment (*P* < 0.05).

**Additional file 3: Table S3.** Clusters of Orthologous Groups of proteins (COG) annotation of DEPs in GE and JS after cold treatment.

**Additional file 4: Table S4.** Gene Ontology (GO) annotation of DEPs in GE and JS after cold treatment.

**Additional file 5: Table S5.** Kyoto Encyclopedia of Genes and Genomes (KEGG) annotation of DEPs in GE and JS after cold treatment.

**Additional file 6: Fig. S1.** Network of interactions among cold-induced DEPs in JS. **a**, DEPs involved in cellular components; **b**, DEPs involved in biological processes; **c**, DEPs involved in molecular functions. The updated platform agriGO v2.0 (http://systemsbiology.cau.edu.cn/agriGOv2/) was used for the construction of interaction among cold-induced DEPs in JS.

**Additional file 7: Table S6.** The list of identified differentially expressed metabolites in GE and JS after cold treatment.

**Additional file 8: Table S7.** The sequences of specific primers used for q-PCR analysis.

**Additional file 9: Table S8.** List of putative candidate proteins in GE and JS after cold treatment

## Data Availability

The datasets generated in this study are included in its additional files, and the raw data is available from the corresponding author on reasonable request.

## Abbreviations

GE: Golden Empress-308
JS: Jia Shi-310
iTRAQ: Isobaric tags for relative and absolute quantification
DEPs: Differentially expressed proteins
ROS: Reactive oxygen species
H_2_O_2_: Hydrogen peroxide
MDA: Malondialdehyde
LC-MS/MS: Liquid chromatography-tandem mass spectrometry
REL: Relative electrolyte leakage
PMSF: Phenylmethanesulfonyl fluoride
EDTA: Ethylenediaminetetraacetic acid
DTT: Dl-dithiothreitol
BSA: Albumin from bovine serum
SDS-PAGE: SDS-polyacrylamide gel electrophoresis
DDA: Data dependent acquisition
GC/MS: Gas Chromatography/mass spectrometry
HCL: Hierarchical cluster analysis
PCA: Principal component analysis
CC: Cellular component
MF: Molecular function
BP: Biological process
GST: Glutathione S-transferase
HSPs: Heat shock proteins
NADPH: Nicotinamide adenine dinucleotide phosphate
PAL: Phenylalanine ammonia-lyase
14–3-3 s: 14–3-3 Proteins
