# Supplementary figures and images for: iTRAQ-based quantitative proteomics analysis of cantaloupe (Cucumis melo var. saccharinus) after cold storage
Source: BMC Genomics. 2020 Jun 3;21:390. doi: 10.1186/s12864-020-06797-3 (PMC7268308; doi:10.1186/s12864-020-06797-3)

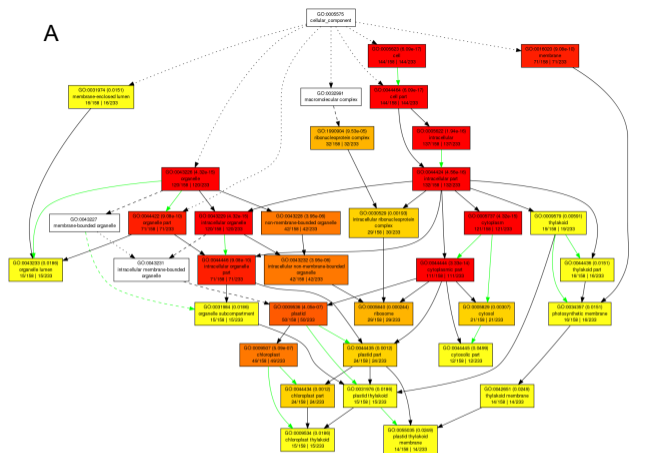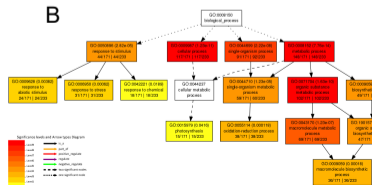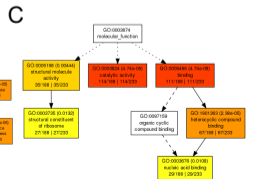

Supplement: Supplementary file 6 — Additional file 6: Fig. S1. Network of interactions among cold-induced DEPs in JS. a, DEPs involved in cellular components; b, DEPs involved in biological processes; c, DEPs involved in molecular functions. The updated platform agriGO v2.0 (http://systemsbiology.cau.edu.cn/agriGOv2/) was used for the construction of interaction among cold-induced DEPs in JS. [file 12864_2020_6797_MOESM6_ESM.pdf]
