# Supplementary material for: iTRAQ-based quantitative proteomics analysis of cantaloupe (Cucumis melo var. saccharinus) after cold storage
Source: BMC Genomics. 2020 Jun 3;21:390. doi: 10.1186/s12864-020-06797-3 (PMC7268308; doi:10.1186/s12864-020-06797-3)
Supplement: Supplementary file 8 — Additional file 8: Table S7. The sequences of specific primers used for q-PCR analysis. [file 12864_2020_6797_MOESM8_ESM.doc]

**Table S7** The sequences of specific primers used for q-PCR analysis

| **ID** | **Description** | **Forward primer (5’3’)** | **Reverse primer (5’3’)** |
| --- | --- | --- | --- |
| LOC103484230 | GAPDH | AAAGACTGGAGAGGTGGAAGAGC | TCAACGGTAGGAACACGGAAAGA |
| XP_008455767.1 | Protein GrpE | TTTCCTTATCCAAATCCACCCT | ATCCTCAGCCTCAGAATCGC |
| XP_008463154.1 | 15.7 KDa heat shock protein peroxisomal | AGTCATTTGGCATTTGGGTG | CTCGGTGTTGTATCTTTGGGTA |
| XP_008444340.1 | Calcyclin-binding protein | CACGGGCTGTTATCACGC | ATTCCTGCCATTGGGTCG |
| XP_008448277.1 | Aldo-keto reductase family 4 member C9-like | TGGCAGCAGAACAAACTACG | TTTCAAGAATATCCCCACCCT |
| XP_008450453.1 | V-type proton ATPase subunit F | TGATGAGGATACGGTTGTTGG | TTAACAGTCGTCTTCGAGTCCAC |
| XP_008460863.1 | Glutathione S-transferase zeta class-like | GTTGTTGCCGACTCTTTTGC | AGAACAGCTAGATTTTGAAGAGGC |
| XP_008454439.1 | Proline iminopeptidase | TAAAAGCGACAGGCAGAGTGA | TGGCTCAATGGGTGGGTAA |
| XP_008465194.1 | 40S ribosomal protein S15-4-like | TACCCACTCTTCAAGGTTCATTC | CTCCAGCCTCCATCGTACTAAT |
| XP_008452081.1 | Polygalacturonase inhibitor-like | GATTGCTGCCATTGGTACTGT | AGTTTGGCTATGGTGGGTTG |
